# Multiple Helminth Infection of the Skin Causes Lymphocyte Hypo-Responsiveness Mediated by Th2 Conditioning of Dermal Myeloid Cells

**DOI:** 10.1371/journal.ppat.1001323

**Published:** 2011-03-17

**Authors:** Peter C. Cook, Sarah A. Aynsley, Joseph D. Turner, Gavin R. Jenkins, Nico Van Rooijen, Mosiuoa Leeto, Frank Brombacher, Adrian P. Mountford

**Affiliations:** 1 Centre for Immunology and Infection, Department of Biology, The University of York, York, United Kingdom; 2 Department of Molecular Cell Biology, Vrjie Universiteit, Amsterdam, The Netherlands; 3 Division of Infectious Immunology, University of Cape Town, Cape Town, South Africa; NIAID/NIH, United States of America

## Abstract

Infection of the mammalian host by schistosome larvae occurs via the skin, although nothing is known about the development of immune responses to multiple exposures of schistosome larvae, and/or their excretory/secretory (E/S) products. Here, we show that multiple (4x) exposures, prior to the onset of egg laying by adult worms, modulate the skin immune response and induce CD4^+^ cell hypo-responsiveness in the draining lymph node, and even modulate the formation of hepatic egg-induced granulomas. Compared to mice exposed to a single infection (1x), dermal cells from multiply infected mice (4x), were less able to support lymph node cell proliferation. Analysis of dermal cells showed that the most abundant in 4x mice were eosinophils (F4/80^+^MHC-II^−^), but they did not impact the ability of antigen presenting cells (APC) to support lymphocyte proliferation to parasite antigen *in vitro*. However, two other cell populations from the dermal site of infection appear to have a critical role. The first comprises arginase-1^+^, Ym-1^+^ alternatively activated macrophage-like cells, and the second are functionally compromised MHC-II^hi^ cells. Through the administration of exogenous IL-12 to multiply infected mice, we show that these suppressive myeloid cell phenotypes form as a consequence of events in the skin, most notably an enrichment of IL-4 and IL-13, likely resulting from an influx of RELMα-expressing eosinophils. We further illustrate that the development of these suppressive dermal cells is dependent upon IL-4Rα signalling. The development of immune hypo-responsiveness to schistosome larvae and their effect on the subsequent response to the immunopathogenic egg is important in appreciating how immune responses to helminth infections are modulated by repeated exposure to the infective early stages of development.

## Author Summary

Schistosomiasis is a major helminth disease that infects more than 200 million people in the tropics. Free-swimming aquatic cercariae infect through the skin after contact with contaminated water, and in endemic areas this can occur frequently. However, nothing is known about how multiple exposures affects innate immunity in the skin, and/or whether it impacts the acquired immune response. Consequently, we have developed an infection model in the mouse to examine the immune response to multiple infections prior to the production of eggs. We show that multiple exposures to schistosome larvae cause lymphocyte hypo-responsiveness, partly mediated by macrophages and dendritic cells from the skin which have a ‘down-regulated’ phenotype and are not able to act as efficient antigen presenting cells (APCs). These regulated APCs are conditioned amongst high levels of the cytokines IL-4 and IL-13 which follow an influx of abundant eosinophils. In the absence of the regulatory APCs, and in the absence of the common receptor chain for IL-4 and IL-13 (*i.e*. IL-4Rα), lymphocyte proliferation is restored. These findings are important in understanding how dermal immune responses are modulated so that we can devise new strategies for vaccine delivery, or the treatment of chronic inflammatory conditions of the skin.

## Introduction

Schistosomiasis is an important tropical disease caused by the parasitic helminth *Schistosoma* and affects 200 million people [1], [2] with a further 779 million at risk of infection [3]. Infection of the host proceeds via the rapid penetration of exposed areas of skin by invasive aquatic cercariae, and people living in endemic areas are likely to repeatedly come into contact with infective cercariae. However, it is not known whether repeated exposure to cercariae affects the development of immune responses in the skin, or responses to later stages of the parasite such as the egg which is the primary agent of Th2 biased immunopathology [2], [4], [5].

The mouse model of schistosome infection provides an important tool with which to examine the early immune response to larval schistosomes. Studies in this model have almost exclusively examined responses to a single infection which are associated with the development of mixed Th1/Th2 responses against normal larvae, although vaccination with live radiation-attenuated cercariae induces a Th1 biased response [6], [7]. Infection elicits an initial neutrophil influx into the skin [8], followed by MHC-II^+^ macrophages (MΦ) and dendritic cells (DC) orchestrated by a cascade of chemokines and pro-inflammatory cytokines [9]. Both MΦ and DC in the dermis take up antigenic excretory/secretory (E/S) material released by invading larvae and are subsequently detected in the skin draining lymph nodes (sdLN) [10] where they have the potential to present parasite antigen to CD4^+^ cells. However, invading larvae and their E/S products can also modulate the dermal immune response [9], [11], [12], [13] and condition DC towards a ‘modulated’ phenotype [14] which prime CD4^+^ cells towards a Th2 phenotype *in vitro* and *in vivo* [15], [16].

A common feature of chronic exposure to helminth infections is the modulation of host immune responses which over time leads to a state of hypo-responsiveness [17], [18], [19]. However, little is known about whether immune responsiveness to helminth infections is determined by the frequency of exposure to infective larvae. In particular it is not known whether multiple exposures to schistosome larvae, and/or their E/S products, deviate innate immune events in the skin, or shape the subsequent development of acquired immune responses [12].

Here, evidence is provided to support the view that multiple exposures of the host to schistosome cercariae modulate the skin immune response and induce hypo-responsiveness of the adaptive response. Two distinct APC populations at the dermal site of infection appear to have a critical role. The first population comprises arginase-1^+^ (Arg-1) Ym-1^+^ AAMΦ-like cells, and the second are functionally compromised MHC-II^hi^ cells. These suppressive myeloid cell phenotypes form as a consequence of events in the skin, most notably an enrichment of IL-4 and IL-13 co-incident with an influx of RELMα-expressing eosinophils. We further show that the development of these suppressive dermal cells is dependent upon IL-4Rα signalling. The importance of immune down-regulation caused by multiple exposures to larvae extends beyond the immediate infection site to distant lymphoid tissues and even modulates the formation of hepatic granulomas elicited by the egg stage of the parasite.

## Results

### Multiple exposures to schistosome cercariae cause CD4^+^ cell hypo-responsiveness in the sdLN

**Figure 1:**
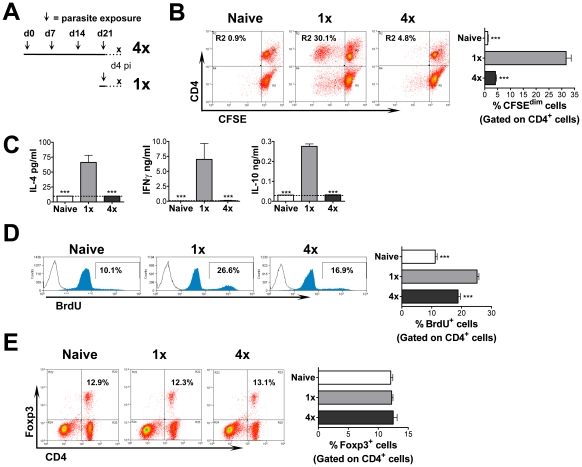
Multiple infections of mice with *S. mansoni* cercariae render CD4^+^ cells in the draining LN hypo-responsive. (A) Infection regime at days 0, 7, 14 and 21 indicated by an arrow (∼100 cercariae per pinna; 50% penetration rate [56]); sdLN sampled at day 4 after the final infection from multiply (4x) and singly (1x) exposed mice. (B) Antigen stimulated *in vitro* proliferation of CFSE-labelled cells from the sdLN of naïve, 1x and 4x infected mice. Representative dot plots show the percentage of CD4^+^ cells that have undergone >1 division and bar chart shows mean values + SEM for 6 mice. (C) Cytokine production from antigen stimulated sdLN cell cultures. Bars show mean + SEM (n = 4 mice); dashed line is lower limit of detection. (D) *In vivo* lymphocyte proliferation measured in naïve, 1x and 4x mice treated with BrdU via the drinking water for 4 days prior to sacrifice. Representative flow histograms of BrdU^+^ cells; bar chart shows mean % BrdU^+^CD4^+^ cells + SEM (n = 7 mice). (E) Representative dot plots showing the proportion of CD4^+^ cells which are Foxp3^+^; bar chart shows mean + SEM (n = 4 mice). P values are of naïve or 4x mice compared to 1x mice. All experiments were repeated at least twice with similar results.

The immune responses in the sdLN of mice exposed to four percutaneous doses (4x) of *S. mansoni* cercariae at weekly intervals were compared with those in mice exposed to a single (1x) infection (Figure 1A). This revealed that following stimulation *in vitro* with larval parasite antigen, CFSE-labelled cells from the sdLN of 4x mice were hypo-responsive in terms of their ability to proliferate and divide, compared to cells from 1x mice (Figure 1B). The hypo-responsive state in 4x mice was particularly marked in the CD4^+^ cell population (4x = 4.8% *cf.* 1x = 30.1%; Figure 1B). Furthermore, while sdLN cells from 1x mice produced abundant antigen-specific IL-4, IFNγ and IL-10, very little or no cytokine was produced by cells from 4x mice (Figure 1C). Hypo-responsiveness in the sdLN was also evident *in vivo* since CD4^+^ cells from 1x mice presented significantly greater uptake of BrdU compared to 4x mice (26.6% *cf.* 16.9%, p<0.001; Figure 1D). However, analysis of the CD4^+^ cell population in the sdLN failed to provide any evidence of expanded Foxp3^+^ regulatory T cell populations (Figure 1E). Hypo-responsiveness was not dependent on the total dose (*i.e.* 4x 100 cercariae), as a single dose of 400 cercariae induced abundant cell proliferation (data not shown). The duration after the initial infection was not a cause of hypo-responsiveness as CD4^+^ cells from 1x mice infected on day 0 and sampled on day 25 (Figure S1A) which failed to proliferate extensively in response to antigen, (Figure S1B), released abundant antigen-driven IFNγ showing that the cells were responsive to antigenic re-stimulation (Figure S1C).

**Figure 2:**
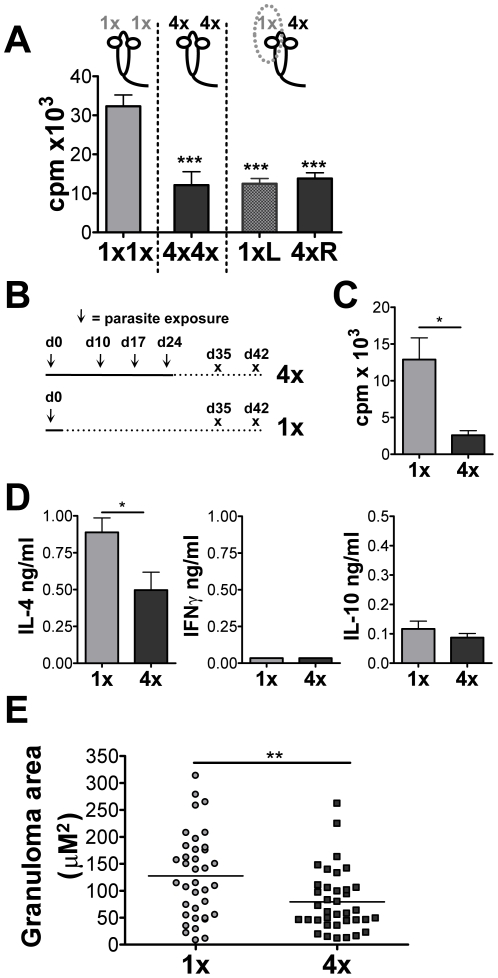
Multiple infections cause systemic immune hypo-responsiveness and down-regulate the size of egg-induced granulomas in the liver. (A) Antigen-specific proliferation of sdLN cells from pinnae exposed to 1x or 4x infections on the left pinnae (1xL) or right pinnae (4xR), or both (1x1x and 4x4x). Results show mean ^3^H-thymidine incorporation (c.p.m.) + SEM (n = 5 mice). (B) Infection regime used to assess the effect of repeated infection on the immune response to mature parasites. (C) Egg-antigen specific proliferation of mesenteric LN cells taken on day 35 from 1x and 4x mice. Bars shows mean ^3^H-thymidine incorporation (c.p.m.) + SEM (n = 5 mice). (D) Egg antigen-specific IL-4, IFNγ and IL-10 production by mesenteric LN cells taken on day 42 from 1x and 4x mice. Bars shows mean cytokine production + SEM (n = 4 mice). (E) Size of hepatic granulomas surrounding single eggs in 1x and 4x mice on day 42; Points are granuloma areas (measured as µm^2^ from H&E stained liver sections + SEM; n = 37 granulomas). P values are of 4x mice compared to 1x mice.

To assess whether hypo-responsiveness was evident in lymphoid tissues distant from the site of infection, mice were exposed to 4x doses of cercariae on the right pinna (4xR) while the left pinna was exposed to only one dose (1xL). Mice exposed to 4x or 1x dose(s) on both pinnae served as controls. As predicted, cells from the sdLN draining 4xR pinnae were hypo-responsive, comparable to mice exposed to 4x doses on both ears (Figure 2A). However, sdLN cells draining the 1xL pinna from the same mouse as 4xR pinna were also hypo-responsive (Figure 2A). This suggests that immune events in the skin exposed to multiple doses of larvae induce hypo-responsiveness even in distant non-draining sdLN (*i.e.* 1xL pinnae) and is not just confined to the local site of infection (*i.e.* 4xR pinnae).

Multiple infections also modulated the immune response after maturation of larvae into adult worms and commencement of oviposition. Five weeks (35 days) after the initial infection (Figure 2B), cells from the mesenteric LN of mice exposed to 4x infections were hypo-responsive in terms of their ability to proliferate *in vitro* to stimulation with SEA compared to cells from mice exposed to a single infection (p<0.05; Figure 2C). Modulation was observed even when a lower infection dose (25 cercariae) was employed (data not shown). At 6 weeks (42 days) after the first infection, 4x mice produced significantly lower levels of IL-4 than cells from 1x mice (p<0.05; Figure 2D). IFNγ was not detectable in either 1x or 4x mice, while only limited amounts of IL-10 were detected, supporting the thesis that multiple infections induce lymphoid hypo-responsiveness. The timing of the infection regime (Figure 2B) ensured that the only source of egg antigens came from the primary and not subsequent infections. Significantly, inflammatory granulomas surrounding embolised eggs in the livers of 4x mice at day 42 were on average 38% smaller in area (µM^2^) than in 1x mice (Figure 2E; p<0.001). This demonstrates that repeated percutaneous exposure to schistosome cercariae causes immune hypo-responsiveness to later developmental stages of the parasite and can down-regulate egg-induced pathology.

### Dermal exudate cells (DEC) from the skin infection site are responsible for mediating CD4^+^ cell hypo-responsiveness

Multiple exposures to schistosome cercariae caused a significant thickening of the skin infection site (Figure S2A). This was largely due to a pronounced infiltrate of inflammatory cells within epidermal and dermal layers (Figure S2B). Therefore, we hypothesised that MHC-II^+^ APC populations within this infiltrate might play an important role in mediating the observed hypo-responsiveness following their migration to the sdLN and presentation of antigen to CD4^+^ lymphocytes [9].

**Figure 3:**
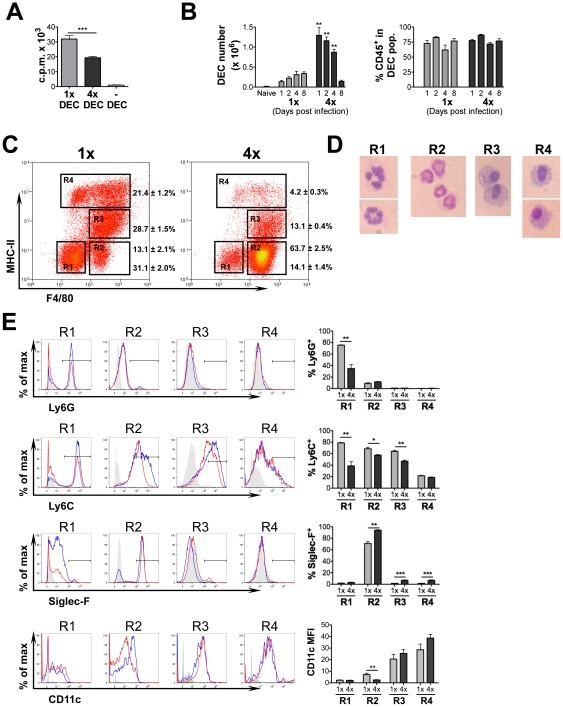
Dermal exudate cells (DEC) from 4x mice are inefficient at supporting antigen-specific CD4^+^ cell proliferation and comprise a large influx of eosinophils but a reduction in MHC-II^+^ cells. (A) DEC recovered from cultured biopsies of 1x and 4x infected skin were co-cultured with purified CD4^+^ T cells from the sdLN of 1x mice and stimulated with parasite antigen. Bars show the mean c.p.m. + SEM (n = 5 DEC samples) and is representative of 4 experiments performed with similar results. (B) Numbers of DEC recovered from naïve, 1x and 4x mice, and the proportion which are CD45^+^ (mean + SEM, n = 6 pinnae/time point). (C) Representative flow cytometry dot plots of DEC recovered on day 4 labelled for MHC-II and F4/80. Values show mean percentage ± SEM of the gated populations R1-R4 (n = 6 mice). (D) Morphology of DEC sorted by MoFlo into R1-R4 on the basis of F4/80 and MHC-II stained with DiffQuick. (E) Representative flow cytometry histogram plots of R1-R4 cell populations labelled with antibodies against Ly6G, Ly6C, SiglecF, and CD11c from 1x (blue) and 4x (red) mice; solid grey plot shows the extent of isotype control antibody staining. Also shown is a bar chart for each marker showing the mean values + SEM for 5 individual mice. Data is representative of at least two experiments.

Skin biopsies from 1x and 4x infected mice were cultured *in vitro* overnight to obtain populations of spontaneously migrating dermal exudate cells (DEC) and then used as APC during co-culture with CD4^+^ cells from the sdLN [20]. The advantage of this isolation technique is that migratory cells can be recovered without having to use a potentially damaging enzymatic digestion step. Significantly, DEC from 1x mice supported much greater levels (>60%) of antigen-specific CD4^+^ cell proliferation than DEC from 4x mice (p<0.001; Figure 3A). Moreover, the superior antigen presenting capacity of 1x DEC was evident with CD4^+^ cells from either 1x or 4x infected mice (data not shown).

### Multiple parasite exposure induces dermal eosinophilia

The total numbers of DEC obtained from 4x mice between days 1 to 4 post-infection were much greater compared to 1x mice (p<0.01; Figure 3B), although the proportions that were CD45^+^ across both groups of mice, at all time points, were similar (60–80%; Figure 3B). Very few (<0.2×10^5^) spontaneously migrating DEC were recovered from naïve mice, indicating that the DEC recovered from 1x and 4x mice represented the infection-induced inflammatory immune cell populations of the skin. DEC consist primarily of neutrophils immediately after infection but an increasing number of DC and MΦ are present during the time that larvae remain in the skin [8], [9], [10]. On the basis of MHC-II and F4/80 expression, four discrete cell populations (R1–R4) were identified (Figure 3C). R1 cells were F4/80^−^ and MHC-II^−^, and comprised a smaller proportion of 4x compared to 1x DEC (p<0.001). The majority of R1 cells were Ly6G^hi^Ly6C^hi^SiglecF^lo^CD11c^lo^ (Figure 3E), suggesting the majority are neutrophils. Cytospins of R1 cells recovered using a MoFlo cell sorter (DakoCytomation) confirmed that morphologically they predominantly consisted of neutrophils (Figure 3D) and that very few lymphocytes were present.

R2 cells (F4/80^+^MHC-II^−^) constituted the majority (>60%) of DEC from 4x mice, and comprised a much greater proportion of the DEC population than from 1x mice (∼5 fold increase; p<0.001; Figure 3C). Moreover, when the numbers of DEC recovered from the two groups of mice (Figure 3B) are taken into account, R2 cells in 4x mice were 15.8-fold more numerous than in 1x mice. R2 cells were the only cells to express high levels of SiglecF (Figure 3E), a marker of eosinophils [21]. R2 cells were also Ly6G^lo^Ly6C^hi^CD11c^lo^, displayed high granularity and cytospins of sorted R2 cells identified them as eosinophils (Figure 3D). The abundance of eosinophils in 4x compared to 1x or naïve mice was confirmed following probing of pinnae sheets with FITC-labelled anti-SiglecF mAb (Figure S3A). Toluidine blue staining of skin sections showed that while the occasional mast cell was detected in the dermis of both naïve and 1x skin, there was a substantial increase in the numbers detected in the skin of 4x mice (p<0.01; Figure S3B & S3C). Mast cells were particularly abundant adjacent to the basement membrane separating the epidermis from the dermis, and many appeared to be degranulating (Figure S3D). However, mast cells were retained in the pinnae and did not migrate during overnight culture as very few IgeR^+^ SiglecF^−^ cells were present in 4x DEC, and only ∼4% were c-kit^+^ (data not shown).

Two further populations of DEC were defined on the basis of differential MHC-II expression: R3 (MHC-II^lo^) and R4 (MHC-II^hi^). R3 cells were also F4/80^+^, while R4 comprised both F4/80^+^ and F4/80^−^ cells (Figure 3C). Both R3 and R4 cells were Ly6G^−^SiglecF^−^ showing this fraction did not contain granulocytes (Figure 3E). Cytospins showed that R3 and R4 cells were largely mononuclear with a large cytoplasm (Figure 3D) and since R3 cells had increased Ly6C expression compared to R4 cells we conclude that R3 cells were likely to be inflammatory MΦ. Whilst both R3 and R4 cells expressed CD11c, the geometric mean fluorescence intensity (MFI) was highest on MHC-II^hi^ R4 cells (Figure 3E), indicating that most R4 cells were DC with high antigen presenting capabilities. Although DEC from 4x mice comprised smaller proportions of both R3 and R4 cells compared to 1x mice, this was presumably due to the massive expansion of R2 eosinophils (53% and 80% decrease respectively; p<0.001; Figure 3C).

The MFI of expression for a number of activation/regulatory factors (i.e. CD40, CD80, CD86, PDL1, PDL2, Fas, and FasL) on R3 and R4 cells was examined, and several were found to be differentially expressed between 1x and 4x mice, and between R3 and R4 cells (Figure S4). CD80, and to a lesser extent CD86, were down-regulated in 4x compared to 1x mice, although the MFI for CD40 was either slightly up-regulated (on R3 cells), or not altered (R4 cells). Together, this suggests that R4 rather than R3 cells are the primary APC population in the DEC population, and that APCs in the skin have reduced expression co-stimulatory molecules following four infections. Both R3 and R4 cells from 4x mice expressed lower MFI of regulatory factor PDL1 but significantly increased PDL2 and Fas (Figure S4). The expression of PDL1 and PDL2 was greater for R4 cells, whilst the MFI for Fas and FasL was much greater on R3 cells; all four of these markers have been associated with regulation of the immune responses but PDL2 is specifically associated with AAMΦ [22].

### The immune environment of multiply-infected mice induces an AAMΦ-like cell population in the skin

**Figure 4:**
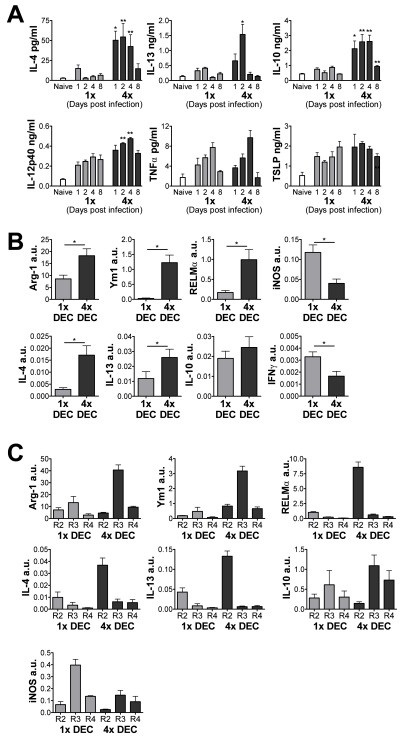
Multiple infections induce a Th2-type cytokine environment in the skin and induce the expression of markers of alternative activation. (A) Cytokine production by skin biopsies from 1x and 4x mice taken at different days post-infection. Bars show mean cytokine + SEM (n = 6). (B) Analysis of mRNA transcript levels from 1x and 4x DEC collected on day 4 post-final infection defined by qRT-PCR. Data are shown in arbitrary units (a.u.) + SEM relative to the expression of GAPDH for each sample (n = 5). (C) Transcript levels of DEC sorted into regions R2-R4 on the basis of F4/80 and MHC-II. Data are means + SEM of 3–4 separate experiments using DEC populations (n = 15 mice). Significances are shown between groups indicated by connector bars, of naïve or 4x mice compared to 1x mice.

The cytokine milieu of the infection site is likely to be important in determining the composition and activation status of the DEC populations. Indeed, supernatants recovered from *in vitro* cultured skin biopsies of infected compared to naive mice contained elevated levels of several soluble immune mediators including TNFα, IL-12/23p40, IL-4, IL-13, IL-10 and TSLP (Figure 4A); IFNγ was not detectable. The supernatants from 4x infected mice were particularly rich in Th2-type cytokines, and over the first 4 days after infection contained 3- to 5-fold increased levels of IL-4 and IL-13 compared to 1x mice, as well as significantly greater quantities of IL-10 (Figure 4A). Though levels of IL-12/23p40 were significantly increased from 4x skin biopsies compared to 1x, it was a less dramatic increase compared to IL-4, IL-13 and IL-10. Furthermore, there were no significant differences between 1x and 4x mice in the levels of TNFα and, perhaps surprisingly, TSLP.

The Th2-like environment in the skin infection site of 4x mice appeared to trigger switching of dermal MΦ from being ‘classically-activated’ (CAMΦ) to ‘alternatively-activated’ as quantitative (q)RT-PCR analysis of mRNA from 4x DEC showed that transcripts for Arg-1, Ym1 and RELMα, which typically characterise AAMΦ [23], [24], [25], were all significantly up-regulated compared to 1x DEC (Figure 4B). Transcripts for IL-4 and IL-13 were also elevated in DEC from 4x mice. Conversely, the expression of iNOS and IFNγ mRNA was significantly lower in 4x compared to 1x DEC.

When DEC were sorted into the R2, R3 and R4 populations as described above (see Figure 3C), only R3 cells (F4/80^+^MHC-II^lo^) from 4x DEC expressed an abundance of Arg-1 and Ym1 transcripts; they did not express RELMα (Figure 4C). This indicates that the R3 fraction comprised a RELMα negative ‘AAMΦ-like’ cell population. In contrast, R3 cells from 1x DEC are likely to be CAMΦ due to their high levels of iNOS transcript combined with low expression of Arg-1, RELMα and Ym1 (Figure 4C). R2 cells (F4/80^+^MHC-II^−^), particularly from 4x DEC, expressed the greatest levels of IL-4 and IL-13 mRNA, and also expressed RELMα transcript. As R2 cells from 4x mice comprised an abundance of eosinophils, this suggests that these RELMα^+^ granulocytes are a source of the Th2-biassed cytokine environment in multiply-infected skin, which in turn may be crucial in driving the formation of the R3 AAMΦ-like cells.

### Both ‘AAMΦ-like’ cells and MHC-II^hi^ APC but not eosinophils from multiply-infected skin are directly responsible for rendering CD4^+^ cells hypo-responsive

**Figure 5:**
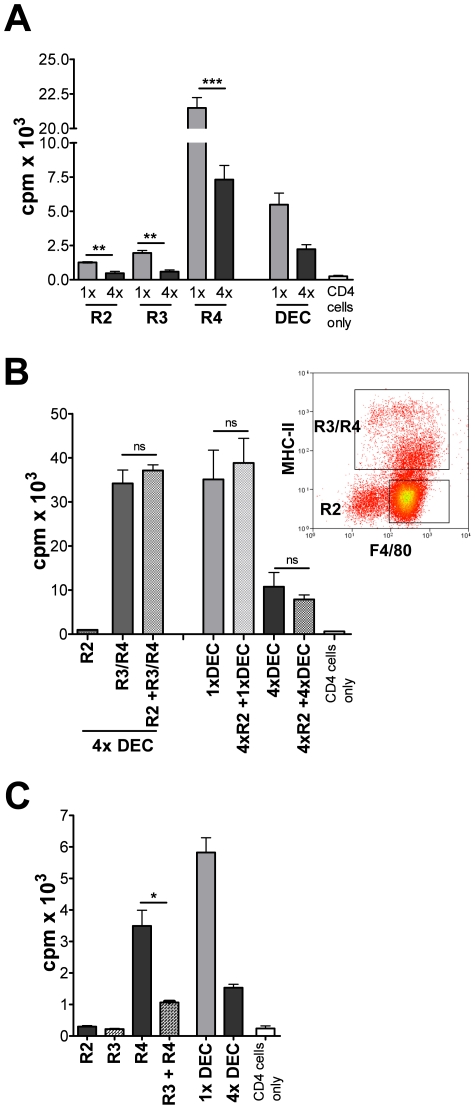
DEC from 4x mice include suppressive and functionally impaired MHC-II^+^ cells, but eosinophils do not directly cause cell hypo-responsiveness. (A) R2, R3 and R4 cells (1×10^4^) from 1x and 4x DEC were co-cultured with purified CD4^+^ cells from 1x sdLN in the presence of parasite antigen. (B) R2 eosinophils (2×10^4^) from 4x DEC were co-cultured with purified CD4^+^ cells from 1x sdLN in the presence of parasite antigen, or together with mixed R3/R4 cells, or unsorted 1x and 4x DEC populations (all 2×10^4^). Bars show CD4^+^ cell proliferation as mean c.p.m. + SEM (n = 5). (C) Sorted R3 and R4 cells from 4x DEC were cultured separately, or combined, with purified CD4^+^ T cells from sdLN of 1x mice. Significances are shown between groups indicated by connector bars. Sorted DEC fractions were pooled from 15–35 mice and bars are mean + SEM of five replicate wells and are representative of 2–3 experiments.

To test which DEC population mediates suppression of sdLN lymphocytes, R2 (eosinophil), R3 (MHC-II^lo^ AAM*Φ*like) and R4 (MHC-II^hi^ DC) cells from 1x or 4x mice were isolated and co-cultured with CD4^+^ cells from 1x infected mice. Whilst R2 and R3 cells from 1x mice induced only low levels of CD4^+^ proliferation, this was even lower when they were obtained from 4x mice in which proliferation was not significantly above that by CD4^+^ cells alone. However, MHC-II^hi^ R4 cells from 1x and 4x DEC were the only cells able to support substantially elevated levels of antigen-specific CD4^+^ cell proliferation (Figure 5A). Strikingly, R4 cells from 4x DEC supported significantly lower (∼3-fold) levels of proliferation compared to R4 cells from 1x mice (p<0.001; Figure 5A). This suggests that the R4 cells from 4x mice, despite expressing high levels of MHC-II, are functionally compromised and that their intrinsic APC potential is impaired.

To establish whether R2 eosinophils from 4x mice modulate the APC potential of MHC-II^+^ cells (*i.e.* R3 and R4 combined), R2 cells were added to MHC-II^+^ cells and used to drive CD4^+^ cell proliferation. The level of CD4^+^ cell proliferation in the presence of R2 cells was similar to that achieved by MHC-II^+^ cells, or unsorted 1x and 4x DEC (Figure 5B). Therefore, the R2 cells do not adversely affect *in vitro* CD4^+^ cell proliferation, either by acting directly on CD4^+^ cells, or by modulating putative APCs present in the R4 population. *In vivo* however, eosinophils may modulate the immune response indirectly as a source of IL-4 and IL-13.

Significantly, addition of R3 (MHC-II^lo^) AAM*Φ*like cells from 4x DEC to co-cultures of R4 (MHC-II^hi^) and CD4^+^ cells suppressed cell proliferation by ∼70% (p<0.05; Figure 5C). Indeed, CD4^+^ proliferation following co-culture with both R3 and R4 cells from 4x mice was reduced to near the level achieved with unsorted 4x DEC and was 82% lower than the level achieved with unsorted 1x DEC. Together, these results show that AAMΦ-like R3 cells from 4x mice are unable to support antigen-specific CD4^+^ proliferation and have a suppressive function on MHC-II^hi^ R4 cells. Thus, R3 but not R2 DEC from multiply infected mice mediate the suppression of CD4^+^ cells from the sdLN.

**Figure 6:**
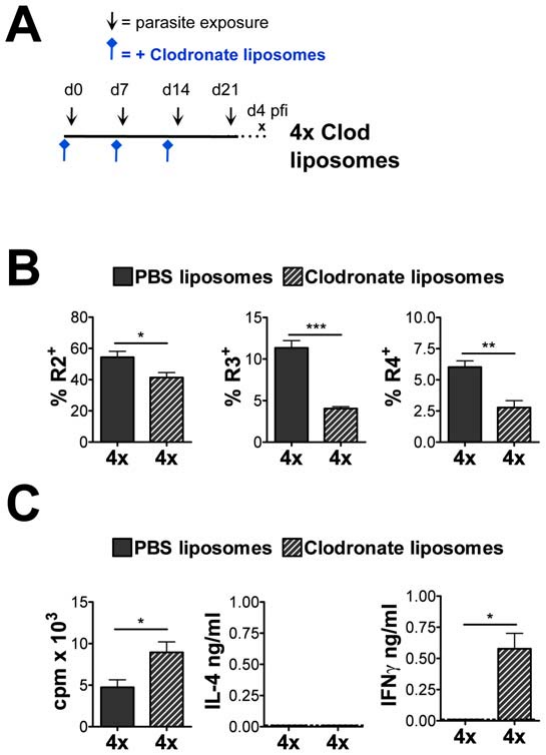
Removal of phagocytic cells through clodronate liposome treatment partially restores lymphocyte responsiveness. (A) PBS- or clodronate-liposomes were given to 4x mice intradermally as indicated prior to infection. (B) Percentage of cells defined by flow cytometry as R2 eosinophils, R3 (MHC-II^lo^ AAM
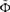
like) and R4 (MHC-II^hi^ DC) recovered from 4x mice that received PBS- or clodronate liposomes. Values are the mean percentage of cells + SEM (n = 4–5 mice). (C) Antigen-specific *in vitro* proliferation and cytokine production by sdLN cells from 1x mice and 4x mice treated with PBS- or clodronate-liposomes. Results show the mean c.p.m. or pg cytokine/ml + SEM (n = 4–5 mice). Significance shown between groups indicated by connector bars, one experiment of two is shown giving similar results.

Removal of phagocytic cells in the skin infection site via clodronate liposome (CL) treatment (Figure 6A), substantially reduced the number of both R3 and R4 DEC from 4x mice, although the numbers of eosinophils was only slightly reduced (Figure 6B). Moreover, the proliferative response of sdLN cells from CL-treated mice was increased compared to PBS-liposome-treated 4x mice (p<0.05) and the production of IFNγ, albeit limited, was also significantly increased (Figure 6C). This further shows that R3 and R4 phagocytes in the skin are compromised in their ability to support lymphocyte responsiveness in the sdLN.

### The cytokine environment in multiply exposed mice causes sdLN hypo-responsiveness and is dependent upon IL-4Rα signalling

**Figure 7:**
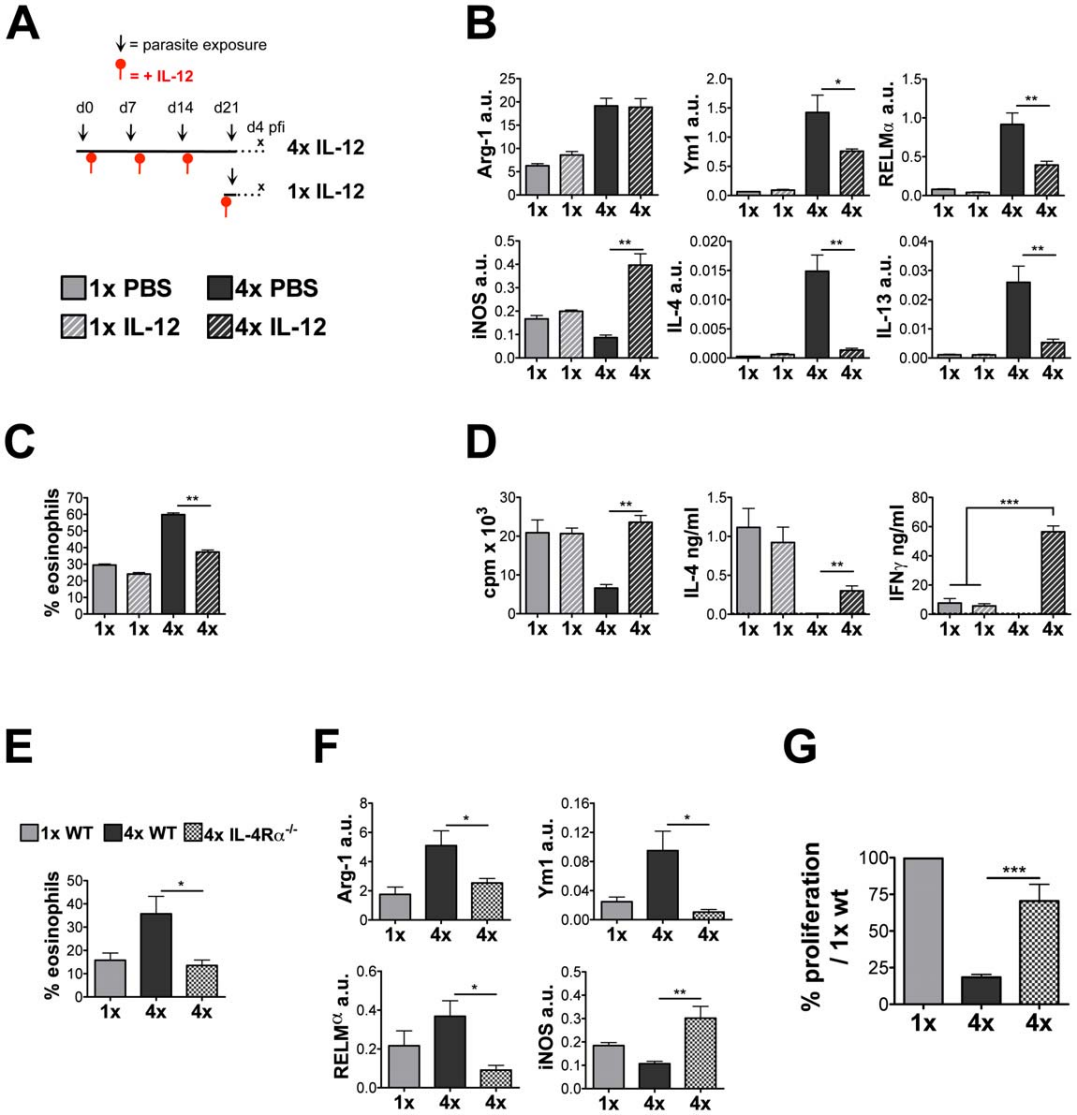
Treatment of 4x mice with rIL-12, or multiple infection of IL-4Rα^−/−^ mice reduces eosinophilia and restores the lymphocyte responsiveness in the sdLN. (A) Treatment regime of rIL-12 administration to 4x mice 2 days after the 1^st^, 2^nd^ and 3^rd^ infection, and 2 days prior to infection for 1x mice. (B) Transcript analysis by qRT-PCR of DEC from PBS or rIL-12 treated 1x and 4x infected mice expressed in arbitrary units (a.u.) relative to GAPDH shown as mean + SEM (n = 4–5). (C) Mean percentage of cells in R2 DEC recovered from rIL-12 or PBS treated 1x and 4x mice + SEM (n = 4–5 mice). (D) Antigen-specific *in vitro* proliferation and cytokine production by sdLN cells from PBS or rIL-12 treated 1x and 4x mice. Results show the mean c.p.m., or pg/ng cytokine/ml, + SEM. (E) Percentage of Siglec-F^+^ and F4/80^+^ cells in DEC recovered from 1x WT, 4x WT and 4x IL-4Rα^−/−^ mice. Bars show mean percentage + SEM of the relevant gated region (n = 5 mice). (F) Transcript analysis of Arg-1, RELMα, Ym-1, and iNOS genes in the total DEC performed by qRT-PCR (n = 5). (G) Antigen-specific *in vitro* proliferation by sdLN cells. Results are shown as the mean percentage change compared to the level of proliferation generated by 1x WT cells + SEM (n = 9–16).

In order to prevent the dominant Th2-type response in the skin of 4x mice and thereby determine whether it drives the formation of modulated APC, recombinant IL-12 (rIL-12) was administered 48 hours after the 1^st^, 2^nd^ and 3^rd^ infections (Figure 7A). DEC from the pinnae of rIL-12 treated 4x mice had much reduced levels of IL-4 and IL-13 transcripts (11- and 5-fold reduction respectively; both p<0.01), but also less RELMα (p<0.01) and Ym1 (p<0.05; Figure 7B). Although the levels of Arg-1 mRNA in 4x DEC were not affected by rIL-12 treatment, levels of iNOS transcript were up-regulated (4.5-fold; p<0.01; Figure 7B). IL-12 treatment had no impact on the number of DEC recovered but it altered the cellular composition of DEC from 4x mice substantially by reducing the proportions of eosinophils (p<0.01; Figure 7C). Moreover, as judged by the expression of iNOS, rIL-12 promotes conditioning of MΦ toward a ‘classically-activated’ status rather than ‘alternatively-activated’ as seen in the PBS-treated control 4x mice. In contrast, the pattern of expression of CD40, CD80, CD86, PD-L1, PD-L2, Fas and FasL by R3 and R4 cell populations (Figure S5) showed that while there were clear differences in expression between R3 and R4 cells obtained from 1x *versus* 4x mice, there were only minor changes in the expression of these molecules between 4x *versus* rIL-12-treated 4x mice. The only significant, albeit slight, changes were up-regulation of CD40, CD80, and PDL2 by R3 cells from rIL-12-treated 4x mice, and Fas by R4 cells. Conversely, PDL2 was down-regulated by R4 cells. Together, this suggests that an obvious marker of ‘modulation’ has not yet been identified.

On the other hand, *in vitro* proliferation of sdLN cells from rIL-12-treated 4x mice was 3.6-fold greater than for cells from sham-treated (PBS) 4x mice (p<0.01), and was similar to that in both groups of 1x mice (Figure 7D). Moreover, the sdLN cells secreted abundant IFNγ (unlike sham-treated 4x mice), which was ∼7.5 fold greater than 1x mice (p<0.001, Figure 7D): delivery of exogenous IL-12 also caused the detection of small quantities of IL-4 compared to sham-treated 4x mice (p<0.01). These data indicate that exogenous IL-12 delivery to the skin prevents the development of sdLN hypo-responsiveness whilst simultaneously modulating dermal eosinophil influx and Th2-conditioning of dermal macrophage populations.

To further investigate the role of dermal cytokines in conditioning DEC phenotype and the generation of lymphocyte hypo-responsiveness, mice deficient for IL-4Rα were exposed to multiple infections. DEC recovered from 4x IL-4Rα^−/−^ mice contained only a small SiglecF^+^ eosinophil population compared to 4x WT mice (p<0.05; Figure 7E), demonstrating that IL-4Rα expression is critical for mediating the influx of eosinophils into the 4x skin infection site. DEC from 4x IL-4Rα^−/−^ mice also had significantly down-regulated levels of mRNA for Arg-1, Ym1 and RELMα but up-regulated levels of iNOS (Figure 7F), confirming that signalling via IL-4Rα is required for the expression of these molecules [23] and the generation of the AAMФ-like population in 4x mice. Our data reveals an essential role for IL-4Rα in the regulation of RELMα, which is confined to the eosinophil population.

The proliferation of sdLN cells from 4x IL-4Rα^−/−^ mice was restored to near the levels achieved by cells from 1x wild-type (WT) mice, clearly showing that IL-4/IL-13 signalling is important in the development of lymphocyte hypo-responsiveness (Figure 7G). Combined, this provides evidence that IL-4Rα^+^ cells contribute towards the generation of lymphocyte hypo-responsiveness and demonstrates that IL-4 and IL-13 cytokine signalling through the IL-4Rα is an important mediator in dampening the immune responses in multiply infected mice.

## Discussion

In this study, we demonstrate that mice multiply-infected with schistosome larvae have increased expression of ‘Th2-associated’ cytokines in the skin-exposure site leading to hypo-responsive lymphoid activity in the sdLN and down-regulated hepatic pathology to schistosome eggs. We conclude that the altered cytokine environment in the infection site of multiply-exposed mice most likely results from an influx of RELMα^+^ eosinophils, which as a source of IL-4 and IL-13 condition dermal MHC-II^+^ myeloid cells with an alternatively-activated and modulated phenotype and makes them inefficient at supporting CD4^+^ lymphocyte activity.

We have established an experimental model of schistosome infection in which the immune response to multiple-exposure with *S. mansoni* larvae can be investigated prior to oviposition and hence in the absence of egg antigens. Mice exposed to four doses of cercariae exhibit lymphocyte hypo-responsiveness supporting earlier studies on multiple infection with the bird schistosome *T. regenti* [26]. The hypo-responsive state extends to sdLN of distant ‘non-exposed’ skin and the mesenteric LN responses at the acute stage of infection leading to the modulation of granulomatous inflammation against eggs in the liver. The down-regulated activity of lymphocytes in the sdLN appears not to involve Foxp3^+^ T_reg_ cells as there was no difference in their frequency in the sdLN of 1x and 4x mice. Rather, it appears to result partially from the development of anergy as *in vitro* responsiveness of sdLN cells can restore lymphocyte activity to a limited extent through the addition of IL-2 (Cook *et al*. MS in preparation).

Modulation of the acquired immune response to chronic schistosome infection is a well accepted immune phenomenon and the presence of eggs and their released antigens are the primary agent [27]. However, whilst modulation of the immune response to multiple schistosome infections has been reported previously [28], [29], parasites were allowed to mature and lay eggs before drug-cure, thereby obscuring the cause of hypo-responsiveness. Here our study clearly demonstrates that multiple exposures of the skin to infective larvae (prior to egg deposition) predispose the host to immune regulation against larval antigens and later developmental stages of the parasite (namely the egg). This suggest that the exposure history of individuals in endemic areas who frequently come into contact with infective parasites [30] is likely to be an important factor in the development of immune responsiveness and hence egg-induced immunopathology.

Typically, chronic helminth infections are associated with the induction of a biased Th2 associated immune response [31], although the response to schistosome parasites prior to egg-laying is thought to comprise a mixed Th1/Th2 phenotype with IFNγ production alongside IL-4 and IL-5 [4]. It is widely accepted that the immune response only becomes dominated by Th2 cells after the start of egg laying [4], although it has also been suggested that exposure to adult worms and their released antigens in the absence of egg antigen can initiate polarisation towards a Th2-phenotype [32]. In light of these observations, we specifically examined whether multiple exposures to infective larvae is conducive to the development of Th2 polarisation. While a Th2 bias was observed in the skin and sdLN in response to non-maturing bird schistosome *T. regenti* larvae [26], we did not observe a Th cell subset bias in the sdLN of mice exposed to 4x doses of *S. mansoni* cercariae since hypo-responsiveness was evident for all the cytokines tested. Nevertheless, analysis of the skin-infection site demonstrated that multiple exposures to cercariae caused dramatically increased levels of IL-4 and IL-13 secretion, as well as increased levels of transcript for these cytokines. Lymphocyte responsiveness was also restored in 4x IL-4Rα^−/−^ mice demonstrating that signalling via IL-4Rα, which is required for both IL-4 and IL-13, has a major influence on the development of hypo-responsiveness.

As the most abundant cell population in the skin after 4x infections were SiglecF^+^ eosinophils, and R2 eosinophils sorted from total DEC expressed abundant mRNA for IL-4 and IL-13, we propose that eosinophils may be the primary source of the copious IL-4 and IL-13 released by 4x skin biopsies. Eosinophils release other pro-Th2/down regulatory molecules such as eosinophil-derived neurotoxin [33], although the expression of RELMα by eosinophils may represent a feedback mechanism to dampen the abundance and potency of Th2-type cytokines [34], [35]. Other tissue resident cells in the skin, such as mast cells and endothelial cells, may release additional polarising mediators such as TSLP [36], [37] but no difference was detected in the levels secreted by the skin of 4x *versus* 1x mice. This implies that TSLP is not likely to be important in conditioning the dermal immune response in our multiple infection model but does not rule out other cytokines such as IL-25 or IL-33 recently described to be important for Th2 induction [38], [39], [40].

It might be argued that the abundance of eosinophils in 4x DEC simply dilutes the number of potential APC accounting for the inability of the total DEC population to support lymphocyte responsiveness. However, R3 an R4 cells from 4x mice in the absence of R2 eosinophils were deficient at supporting lymphocyte proliferation. Moreover, we found no evidence that purified eosinophils from 4x DEC directly or indirectly down-regulate *in vitro* lymphocyte responses supported by putative APCs. Instead, eosinophils may contribute towards the development of hypo-responsiveness in our infection model by conditioning dermal cells that subsequently traffic to the sdLN where they mediate the extent of the acquired immune response.

MФ are especially sensitive to high levels of IL-4 and IL-13 and become ‘alternatively-activated’ [41]. In fact, AAMΦ-like cells (R3) are a major constituent of the DEC population of 4x mice, and while most studies on AAMΦ elicited by helminth infections have been on cells in the intestines, lungs or peritoneal cavity [24], [25], [34], [41], [42], [43], [44], [45], our study is the first to report their presence in the skin. Conventional AAMΦ observed following helminth infection are IL-4/IL-13-dependent, and analyses of the DEC mRNA transcript levels demonstrated that the AAMΦ-like population was absent in 4x IL-4Rα^−/−^ mice. However, although RELMα has been previously thought to be a defining characteristic of AAMΦ [41], we note that our AAMΦ-like cell population obtained from the skin does not express abundant RELMα and may represent a tissue-specific sub-population of MФ. The MФ population in 4x IL-4Rα^−/−^ mice instead displayed a CAMΦ phenotype accompanied by increased levels of MHC-II. AAMФ are required for the induction of protective memory Th2 responses against gut helminths [46], possibly via increased Ym1 [47]. However, sdLN cells from our repeatedly infected mice displayed down-regulated Th2 cytokine production suggesting that the AAMΦ-like cells in our infection model are not involved in the promotion of Th2 responses. AAMΦ-like cells may be required for eosinophil recruitment [48]. Indeed, 4x mice treated with clodronate liposomes to deplete phagocytic cells had a reduced influx of eosinophils, although the remaining population was still substantial in number.

The AAMΦ-like cells revealed in our studies were functionally suppressive and mediated hypo-responsiveness of sdLN cells. They expressed arginase and Ym1 but not RELMα transcript which may highlight the heterogeneity of AAMΦ depending upon their tissue location (*i.e.* the skin), and/or reflect a ‘wound healing’ phenotype defined as M2c MΦ within a ‘colour wheel’ of immune function [49], [50]. The sorted R3 AAMΦ-like DEC population in 4x mice down-regulated CD4^+^ T cell responses supported by MHC-II^hi^ APCs, a feature previously described for conventional AAMΦ [51]. Removal of the dermal AAMΦ-like population by clodronate liposomes also lead to significant increases in the proliferative responses of sdLN cells. Therefore, we conclude that irrespective of their precise classification, the AAMΦ-like cells in our model are an important component causing down-regulation of lymphocyte proliferation and cytokine production.

In addition to the AAMΦ-like cells, we show that dermal MHC-II^hi^ APCs from 4x mice were less efficient at supporting the lymphocyte response compared to R4 cells from 1x mice on a ‘cell-to-cell’ basis (Figure 5A). The mechanism by which these cells were functionally impaired is unclear and may be related to decreased expression of MHC-II, CD80 or CD86, or elevated expression of PDL2 and Fas. However, after IL-12 treatment of 4x mice, the expression of activation *versus* regulatory factors was not markedly altered, suggesting that other as yet un-identified molecule(s) play a critical role. Expression of Arg-1 and Ym1 transcripts, indicative of an ‘alternatively-activated’ population, were greater in MHC-II^hi^ DEC from 4x compared to 1x mice (Figure 4C) and, although expression of these markers by DC has been previously identified [25], [52], it is not known what impact this has on their ability to support lymphocyte responsiveness. The large quantities of IL-10 released by 4x skin biopsies may impair DC activation of CD4^+^ cells as IL-10 can generate tolerogenic DC [53]. Furthermore, we speculate that since clodronate treatment did not completely ablate the R4 cell population, the remaining cells represent modulated APCs such as Langerhan's cells which are not affected by clodronate treatment [54]. This could explain why the sdLN response of CL-treated mice was not restored to the levels seen in 1x mice.

The ability of APCs, and DC in particular, to support T cell proliferation needs to also be viewed in the context of how they are stimulated by parasite specific antigens.

Like schistosome egg antigens [55], molecules released by the invading cercariae (named 0–3hRP) stimulate limited maturation of bone marrow-derived DC [14] which drive Th2 responses both *in vitro* and *in vivo* [15]. Recognition of 0–3hRP by potential APCs occurs via TLRs [16] and/or C-type lectin receptors, such as the mannose receptor (Paveley *et al.*, MS in preparation), drives arginase production by cultured DC and MΦ, suggestive of alternative activation [10]. Repeated exposure to these cercarial complexes may accentuate their properties and so interfere with the ability of APCs to support T cell responsiveness.

This study provides evidence that the skin-infection site of mice frequently exposed to an infectious pathogen is important in determining the nature of subsequent acquired immune responses. Formation of AAMΦ-like and modulated MHC-II^hi^ cells in the skin represent previously unknown mechanisms by which the host immune response limits harmful pathology to subsequent doses of an infectious agent. In the context of schistosome infection, our studies show that exposure to larvae and their antigens, prior to the arrival of eggs, can initiate immune hypo-responsiveness against different stages of the parasite. This has important consequences in the development of future vaccination strategies but also has implications in the prevention of immune-related pathology to embolised eggs.

## Materials and Methods

### Ethics statement

All experiments were carried out in accordance with UK Animal's Scientific Procedures Act 1986 and with approval of the University of York Ethics Committee.

### Mice, parasites and *in vivo* treatment regimes

Female C57BL/6 mice were bred in house at the University of York and used aged 8–12 weeks. IL-4Rα^−/−^ on a BALB/c background were kindly provided by Dr F. Brombacher and experiments were performed at the University of Cape Town. A Puerto Rican strain of *S. mansoni* was maintained by routine passage through outbred NMR-I mice and *Biomphalaria glabrata* snails maintained at University of York. Mice were exposed to either a single (1x), or four (4x) dose(s) of 100 *S. mansoni* cercariae via each pinna [56] at weekly intervals between day 0 and 21 (Figure 1A). Penetration rates were approximately 50%, therefore, the combined infection dose per mouse after 4x infections was approximately 400 larvae.

To assess *in vivo* cell proliferation, mice were given 5-Bromo-2′deoxyuridine (BrdU; Sigma-Aldrich) via the drinking water (0.8 mg/ml), for four days prior to sdLN removal. To ablate phagocytic cells from the skin infection site, clodronate liposomes (CL), or PBS-loaded liposomes in 10 ml, were administered intradermally to the pinnae 72 hours prior to the 1^st^, 2^nd^ and 3^rd^ infection. Liposomes were prepared as previously described by Dr N. van Rooijen [57] using phosphatidylcholine (LIPOID E PC; Lipoid GmbH) and cholesterol (Sigma). Clodronate (Cl2MDP) was a gift of Roche Diagnostics GmbH, (Mannheim, Germany). In some experiments, rIL-12 (gift of Dr S. Wolf, Genetics Institute, Cambridge, MA USA), or an equivalent volume of PBS (10 µl), was delivered intradermally into the pinnae and intraperitoneally (0.25 µg and 0.2 µg, respectively) 48 hours after the 1^st^, 2^nd^, and 3^rd^ *S. mansoni* infection (Figure 6A). In mice receiving a single infection, rIL-12 was given once 48 hrs prior to infection.

### *In vitro* culture of sdLN cells

Cells from the sdLN were cultured (1×10^6^ cells/ml) for 4 days in RPMI-1640 containing 10% low endotoxin FCS (Harlan Sera labs), 2 mM L-Glutamine, 200 U/ml penicillin, 100 µg/ml streptomycin and 50 µM 2-ME (all Invitrogen), in the presence of soluble Ag prepared from larval schistosomes (50 µg/ml) [9] and cell proliferation measured by [^3^H]thymidine incorporation (18.5 kBq/well; Amersham Biosciences)[20]. Alternatively, sdLN cells were labelled with 3 µM CFSE (Molecular Probes) for 15 min, washed and after chase incubation, cultured for 3 days with or without Ag. Culture supernatants were collected at 72 hr for cytokine detection by ELISA.

### Analysis of the skin infection site and recovery of DEC

Inflammation of pinnae was measured using a dial gauge micrometer (Mitutoyo, Japan). For histological analysis, pinnae were removed, fixed in 10% neutral buffered formal saline, wax-embedded, sectioned at 5 µm and stained with Hematoxylin and Eosin, or Toluidine Blue (Department of Veterinary Pathology, University of Liverpool, UK). Pinnae sheets separated from the central cartilage were incubated with optimal concentrations of anti-Siglec-F FITC labelled antibody (BD Pharmingen) prior to mounting and imaging using a Zeiss confocal LSM 510 meta microscope.

For the recovery of dermal exudate cells (DEC), freshly excised pinnae were split in two along the central cartilage, and cultured *in vitro* for 18 hr in the absence of added Ag as described previously [9], [56]. DEC were then recovered and prepared for phenotyping, or cell sorting as below. Culture supernatants from the skin biopsies were stored at −20°C for cytokine detection by ELISA.

### Immune responsiveness at sites distant to the multiple infection site

To assess the immune response at skin sites distant to the site of infection, mice were infected at weekly intervals as above with 100 cercariae via the right pinna. At the 4^th^ infection, both the right ( = 4xR) and the previously uninfected left (1xL) pinnae were infected and immune assays performed on the pinnae (*i.e.* 4xR and 1xL) and their respective sdLN 4 days later.

To assess the effect of multiple infections on immune responses to later stages of parasite development, one group of mice (denoted as 1x) were exposed to 100 cercariae on the pinnae on day 0, and then sacrificed at days 35 or 42, by which time adult worms had matured and commenced egg deposition. A parallel group of mice (denoted as 4x) was similarly infected on day 0, and again on days 10, 17, and 24, before sacrifice on days 35 or 42. The mesenteric LN were removed and cultured as for sdLN but the parasite Ag was soluble egg antigen (SEA). Lymphocyte proliferation and cytokine production from LN cell cultures were measured as above. The liver was wax-embedded, sectioned at 5 µm and stained with Hematoxylin and Eosin; granuloma areas surrounding individual eggs were determined using AxioVision 4.3 (Zeiss UK Ltd) and expressed as mm^2^.

### Cytokine ELISA

ELISAs were used to quantify IL-12/23p40, IL-6, IL-4, and IFNγ in the pinnae biopsy and sdLN culture supernatants as previously described [9]. IL-13 and TSLP were measured by DuoSet ELISA kit (R&D Systems), TNFα and IL-10 by Cytoset (Invitrogen).

### Flow cytometry and MoFlo cell sorting

DEC were blocked with anti-CD16/32 mAb (BD Pharmingen) in PBS (supplemented with 1% FCS & 5 mM EDTA) and subsequently labelled with the following conjugated antibodies; F4/80 FITC, Pacific Blue or PE-Cy7 (BM8), CD11c APC-eFlour® 780 (N418), SiglecF PE (E50-2440), Ly6C APC (AL-21), Ly6G PerCP-Cy5.5 (1A8), CD40 PE (3/23), CD80 APC (16-10A1), CD86 PerCP-Cy5.5 (GL1), and I-A^b^ biotin or FITC (28-16-8S), PDL1 biotin (MIH5), PDL2 PE (122), Fas PE (15A7), FasL biotin (MFL3) (Ab from BD Pharmingen, BioLegend, Caltag Medsystems, eBioscience and GeneTex Inc.). Biotin conjugated antibodies were probed with streptavidin APC (Caltag Medsystems). Cells isolated from the sdLN were stained CD4 FITC (RM4-5), Foxp3 PE (FJK-16s). BrdU staining was performed using FITC-conjugated anti-BrdU with DNase according to manufacturer's instructions (BD Pharmingen). All antibody concentrations were optimised and labelling performed alongside relevant isotype controls. Flow cytometric acquisition was performed using a Cyan ADP analyser and analysed with Summit v4.3 (DakoCytomation) or FlowJo software (Tree Star, Inc.). DEC labelled with F4/80 and I-A^b^ mAb were separated using a MoFlo cell sorter (Dako) revealing 4 populations of live cells gated to give purity >70–90%. Cytospins of the cell fractions (Cytospin 2, Shandon) were stained with Diff-Quik (Dade) to determine cell morphology.

### CD4^+^ cell co-culture with unsorted and sorted DEC populations

CD4^+^ cells from 1x and 4x infected mice were isolated via negative selection (MACS LS column; Miltenyi Biotec); cell purities were >95%. CD4^+^ cells (5×10^4^ cells) were co-cultured with unsorted DEC (2×10^4^ cells), or sorted R2, R3 and R4 DEC (1 or 2×10^4^ cells), for 4 days in round-bottom 96 well plates in the presence of soluble larval parasite Ag (50 µg/ml) [20]. Cell proliferation and cytokine analysis was performed as described above.

### Real time quantitative PCR

Cells were re-suspended in TRIzol (Invitrogen) and total RNA extracted. After synthesis of cDNA using Superscript III DNA polymerase (Invitrogen), various genes were analysed by qRT-PCR (ABI PRISM 7000; Applied Biosystems) using Taqman probes (Sigma-Aldrich). The relative expression of each gene was normalised to the values for the GAPDH before statistical analysis. The primer pairs and probes were; Arg-1:

5′-TCACCTGAGCTTTGATGTCG, 5′-CTGAAAGGAGCCCTGTCTTG,

Probe 5′-TTCTGGGAGGCCTATCTTACAGAGAAGGTCTCTAC,

RELMα:

5′-TGCTGGGATGACTGCTACTG, 5′-CTGGGTTCTCCACCTCTTCA,

Probe 5′-CAAGATCCACAGGCAAAGCCACAA,

Ym1:

5′-CTCAATATACACAGTGCAAGTTG, 5′TGGGATTCAATTTAGGAAAGTTCA,

Probe TCCACAGTGCATTCTGCATCATGCT,

iNOS:

5′-CTGCATGGACCAGTATAAGG, 5′-CTAAGCATGAACAGAGATTTCTTC, Probe: 5′-AGTCTGCCCATTGCTG,

IL-4:

5′-CTCACAGCAACGAAGAACAC,

5′-TAAATAAAATATGCGAAGCACCTTG,

Probe 5′-AAGCCCTACAGACGAGC,

IL-10:

5′-GGTCTTGGGAAGAGAAACCAG,

5′-GCCACAGTTTTCAGGGATGA,

Probe 5′-CTTTGATGATCATTCCTGCAGCAGCTC,

IL-13: 5′-TTATTGAGGAGCTGAGCAAC, 5′-GAGATGTTGGTCAGGGAATC, Probe 5′-TACACAGAACCCGCCAG,

IFNγ: 5′-GCGTCATTGAATCACACCTG, 5′-TGAGCTCATTGAATGCTTGG, Probe 5′-TTGAGGTCAACAACCCACAGGTCCA,

GAPDH: 5′-CCATGTTTGTGATGGGTGTG, 5′-CCTTCCACAATGCCAAAGTT, Probe 5′-CATCCTGCACCACCAACTGCTTAGC.

### Statistics

Statistical analysis was performed using Student's t test, or one-way ANOVA. Values of *p*<0.05 were considered significant: * p<0.05; ** p<0.01; *** p<0.001.

## Supporting Information

Figure S1 Hypo-responsiveness caused by multiple infections is not due to duration after the first infection. (A) Infection regime at days 0, 7, 14 and 21 indicated by an arrow (∼100 cercariae per pinna), sdLN sampled at day 4 or day 25 after single infection (1x and 1x day 25 respectively) or day 4 after multiple infection (4x). (B) Antigen stimulated *in vitro* proliferation of CFSE-labelled cells from the sdLN of naïve, 1x, 4x, and 1x day 25 infected mice. Bar graph shows the mean + SEM of percentage of CD4^+^ cells that have undergone >1 division (n = 6 mice). (C) IFNγ production from antigen stimulated sdLN cell cultures. Bars show mean + SEM (n = 4 mice); dashed line is lower limit of detection. All experiments were repeated at least twice with similar results.
(0.15 MB TIF)

Figure S2 Multiple exposures to infective cercariae cause inflammation of the skin infection site. (A) Pinnae thickness of naïve, 1x and 4x mice on days post-final infection are expressed as mm + SEM (n = 6 pinnae). One of three experiments is shown. (B) Representative transverse sections through pinnae stained with H∧E: epidermis, D: dermis, C: cartilage. P values are of 4x pinnae compared to 1x cohorts.
(2.25 MB TIF)

Figure S3 Multiple doses of infective parasites cause the recruitment of SiglecF^+^ eosinophils and mast cells. (A) Pinnae from naïve, 1x and 4x mice were isolated and tissue sheets labelled with anti-Siglec-F FITC and imaged using a Zeiss confocal LSM 510 Meta microscope. (B) Transverse sections of pinnae stained for mast cells with Toluidine blue (cells stained purple) and (C) total numbers of mast cells counted per field of view (n = 20). (D) High power images (x64) of mast cells adjacent to the membrane separating the epidermis from the dermis, and in the process of degranulation. P values are of 4x pinnae compared to 1x cohorts.
(7.65 MB TIF)

Figure S4 Multiple exposures to infective cercariae induces changes in the expression of co-stimulatory and regulatory factors on R3 and R4 DEC. Representative flow cytometry histogram plots of R3 and R4 DEC populations labelled with antibodies against CD40, CD80, CD86, PD-L1, PD-L2, Fas and FasL from 1x (blue) and 4x (red) mice; solid grey plot shows the extent of isotype control antibody staining. Also shown is a bar chart showing the MFI expression for each marker as mean values + SEM for 5 individual mice.
(1.03 MB TIF)

Figure S5 Administration of rIL-12 does not markedly alter the expression of co-stimulatory and regulatory factors on R3 and R4 DEC from 4x mice. Representative flow cytometry histogram plots of R3 and R4 DEC populations labelled with antibodies against CD40, CD80, CD86, PD-L1, PD-L2, Fas and FasL from 1x (blue), 4x (red) and rIL-12-treated 4x mice (green); solid grey plot shows the extent of isotype control antibody staining. Also shown is a bar chart showing the MFI expression for each marker given as mean values + SEM for 5 individual mice.
(1.33 MB TIF)
