# Supplementary figures and images for: Multiple Helminth Infection of the Skin Causes Lymphocyte Hypo-Responsiveness Mediated by Th2 Conditioning of Dermal Myeloid Cells
Source: PLoS Pathog. 2011 Mar 17;7(3):e1001323. doi: 10.1371/journal.ppat.1001323 (PMC3060168; doi:10.1371/journal.ppat.1001323)

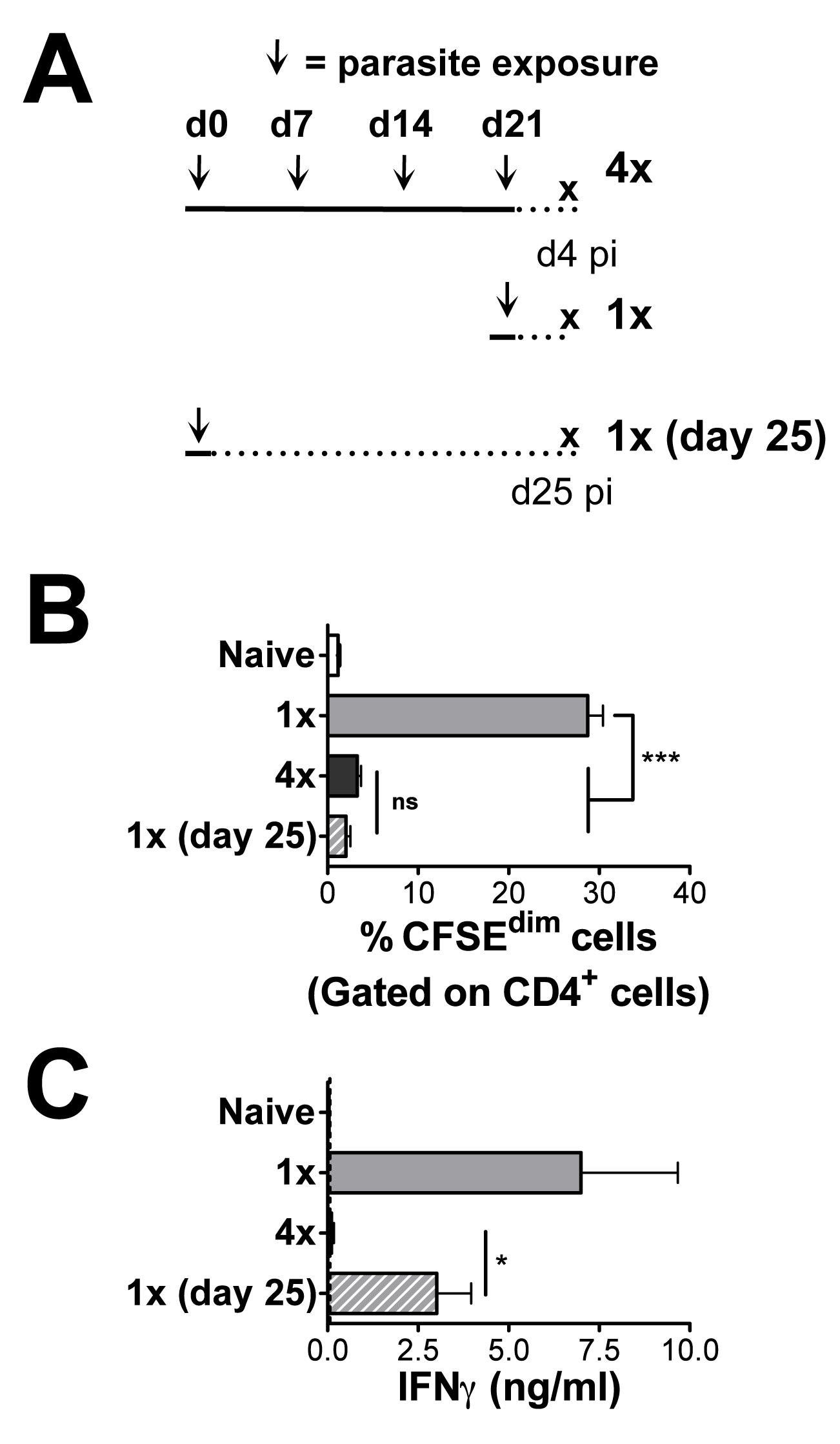

Supplement: Figure S1 — Hypo-responsiveness caused by multiple infections is not due to duration after the first infection. (A) Infection regime at days 0, 7, 14 and 21 indicated by an arrow (∼100 cercariae per pinna), sdLN sampled at day 4 or day 25 after single infection (1x and 1x day 25 respectively) or day 4 after multiple infection (4x). (B) Antigen stimulated in vitro proliferation of CFSE-labelled cells from the sdLN of naïve, 1x, 4x, and 1x day 25 infected mice. Bar graph shows the mean + SEM of percentage of CD4+ cells that have undergone >1 division (n = 6 mice). (C) IFNγ production from antigen stimulated sdLN cell cultures. Bars show mean + SEM (n = 4 mice); dashed line is lower limit of detection. All experiments were repeated at least twice with similar results. (0.15 MB TIF) [file ppat.1001323.s001.tif]

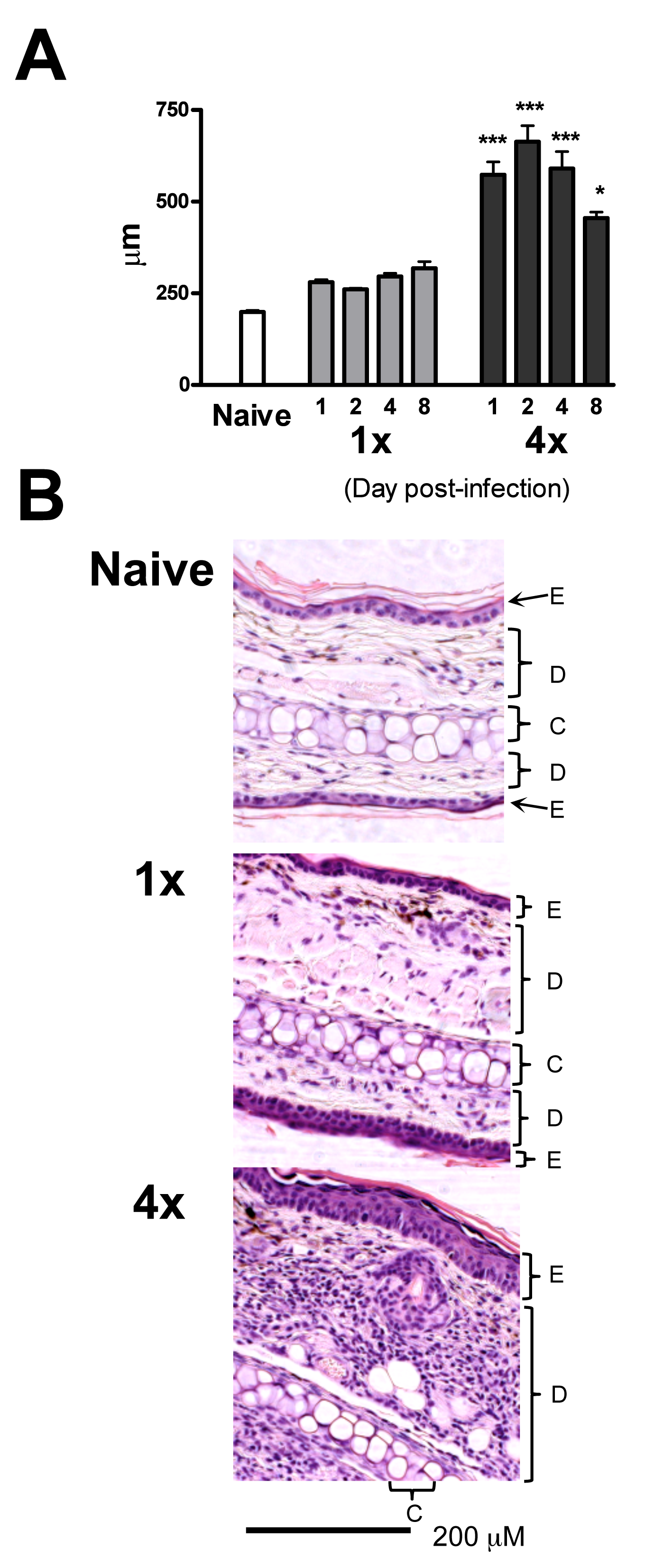

Supplement: Figure S2 — Multiple exposures to infective cercariae cause inflammation of the skin infection site. (A) Pinnae thickness of naïve, 1x and 4x mice on days post-final infection are expressed as mm + SEM (n = 6 pinnae). One of three experiments is shown. (B) Representative transverse sections through pinnae stained with H∧E: epidermis, D: dermis, C: cartilage. P values are of 4x pinnae compared to 1x cohorts. (2.25 MB TIF) [file ppat.1001323.s002.tif]

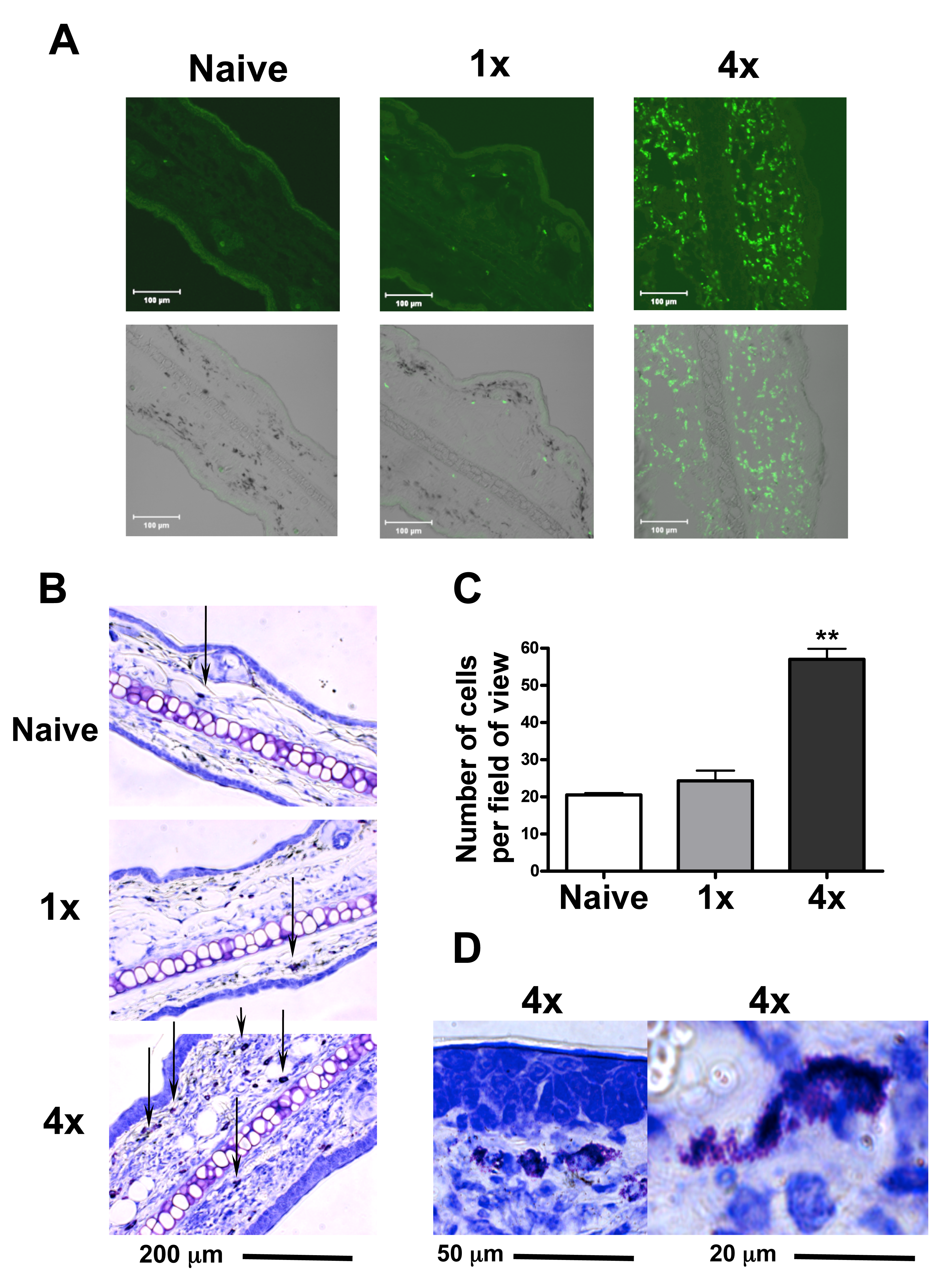

Supplement: Figure S3 — Multiple doses of infective parasites cause the recruitment of SiglecF+ eosinophils and mast cells. (A) Pinnae from naïve, 1x and 4x mice were isolated and tissue sheets labelled with anti-Siglec-F FITC and imaged using a Zeiss confocal LSM 510 Meta microscope. (B) Transverse sections of pinnae stained for mast cells with Toluidine blue (cells stained purple) and (C) total numbers of mast cells counted per field of view (n = 20). (D) High power images (x64) of mast cells adjacent to the membrane separating the epidermis from the dermis, and in the process of degranulation. P values are of 4x pinnae compared to 1x cohorts. (7.65 MB TIF) [file ppat.1001323.s003.tif]

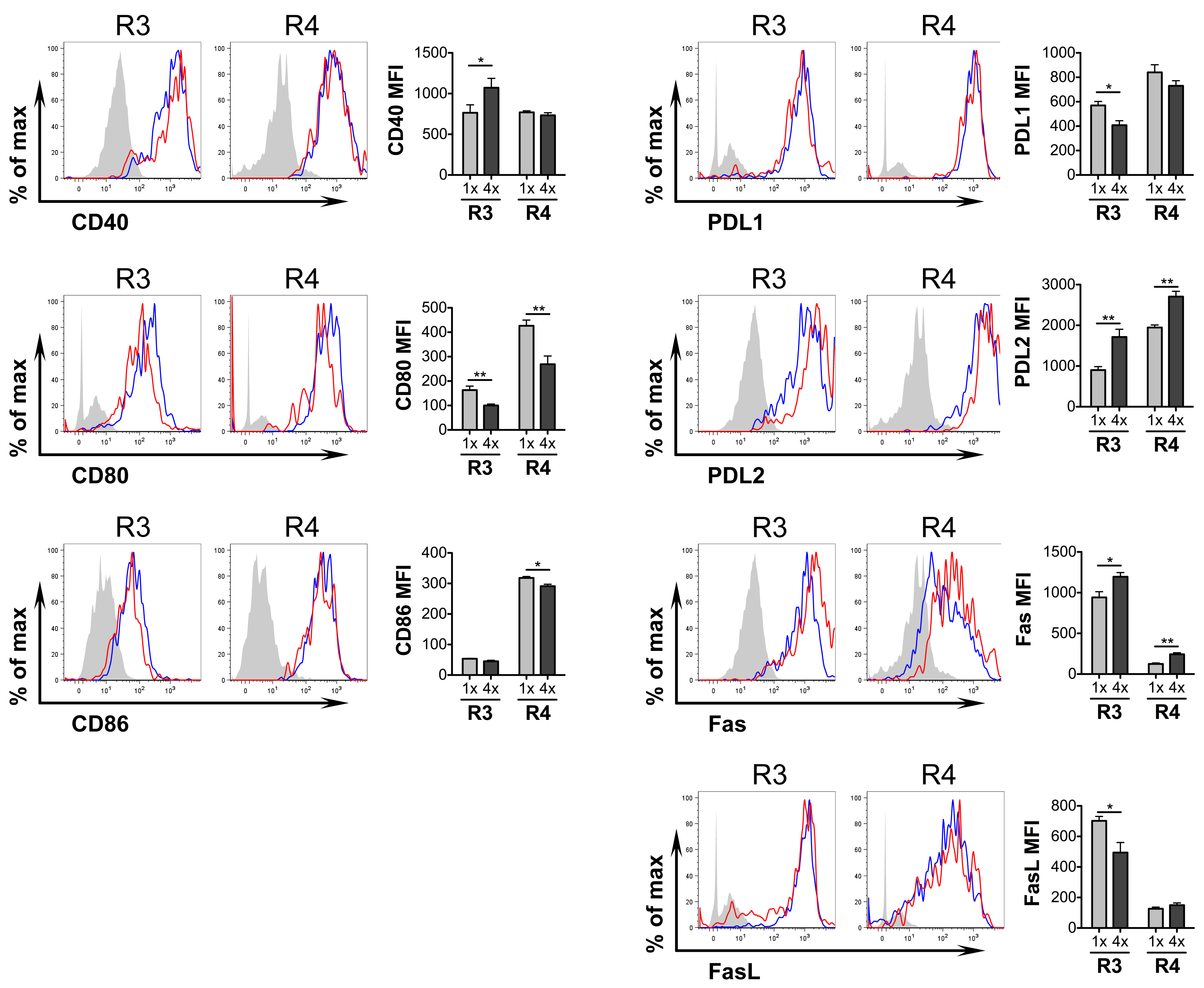

Supplement: Figure S4 — Multiple exposures to infective cercariae induces changes in the expression of co-stimulatory and regulatory factors on R3 and R4 DEC. Representative flow cytometry histogram plots of R3 and R4 DEC populations labelled with antibodies against CD40, CD80, CD86, PD-L1, PD-L2, Fas and FasL from 1x (blue) and 4x (red) mice; solid grey plot shows the extent of isotype control antibody staining. Also shown is a bar chart showing the MFI expression for each marker as mean values + SEM for 5 individual mice. (1.03 MB TIF) [file ppat.1001323.s004.tif]

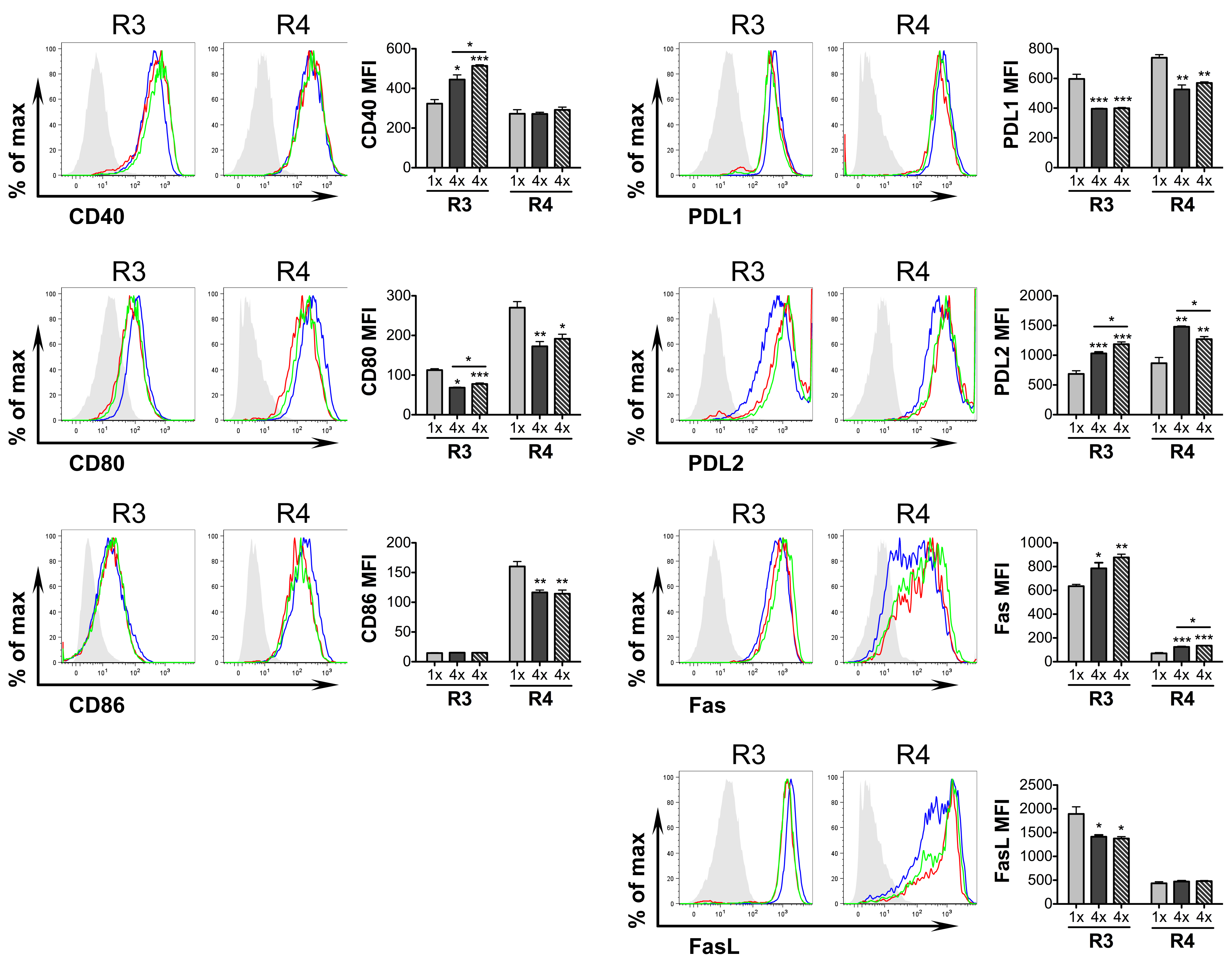

Supplement: Figure S5 — Administration of rIL-12 does not markedly alter the expression of co-stimulatory and regulatory factors on R3 and R4 DEC from 4x mice. Representative flow cytometry histogram plots of R3 and R4 DEC populations labelled with antibodies against CD40, CD80, CD86, PD-L1, PD-L2, Fas and FasL from 1x (blue), 4x (red) and rIL-12-treated 4x mice (green); solid grey plot shows the extent of isotype control antibody staining. Also shown is a bar chart showing the MFI expression for each marker given as mean values + SEM for 5 individual mice. (1.33 MB TIF) [file ppat.1001323.s005.tif]
